# Anatomical Relationship of the Mylohyoid Ridge, Lingual Concavity, and Mandibular Canal: A Retrospective CBCT Study †

**DOI:** 10.3390/diagnostics15172233

**Published:** 2025-09-03

**Authors:** Selva Sen, Melike Nur Girit, Gamze Ansen, Kadriye Betul Pence, Neslihan Yuzbasioglu

**Affiliations:** 1 School of Dentistry, Istanbul Galata University, Istanbul 34420, Türkiye; 2 Graduate School of Health Sciences, Istanbul Medipol University, Istanbul 34810, Türkiye; melike.girit@medipol.edu.tr (M.N.G.); kbpence@medipol.edu.tr (K.B.P.); 3 Department of Anatomy, School of Medicine, Istanbul Medipol University, Istanbul 34810, Türkiye; ncankara@medipol.edu.tr; 4 Department of Anatomy, International School of Medicine, Istanbul Medipol University, Istanbul 34810, Türkiye; gansen@medipol.edu.tr

**Keywords:** mylohyoid ridge, lingual concavity, mandibular canal, submandibular fossa, CBCT

## Abstract

**Background/Objective**: This study aimed to determine the position of the mylohyoid ridge (MR) and lingual concavity (LC) in the mandible and their relationship with the mandibular canal (MC) and submandibular fossa, to provide anatomical guidance for surgical procedures in this region. **Methods**: A retrospective analysis was performed on cone beam computed tomography (CBCT) scans from 200 adult patients who had undergone imaging for dental treatment. On cross-sectional images at the level between the first and second molar roots, the following measurements were obtained: LC horizontal depth, LC height, LC depth, MR height, MR depth, and the distances from MR to MC (MR–MC) and LC to MC (LC–MC). **Results**: Mean values were: LC horizontal depth: 3.72 ± 0.90 mm, LC height: 11.74 ± 2.01 mm, LC depth: 12.54 ± 3.03 mm, MR height: 17.66 ± 2.60 mm, MR depth: 6.87 ± 2.38 mm, MR-MC: 8.30 ± 2.00 mm, and LC-MC: 3.72 ± 0.87 mm. All parameters were symmetrical between the right and left sides of the mandible, with no significant sex differences. The positions of the MR and LC were related to each other, and the position of the MC was related to the positions of the LC and MR. There was no correlation between the horizontal depth of the LC and the position of the MR, LC and MC. **Conclusions**: The vertical positions of the MR and LC are related to each other and MC. Therefore, it can be used as a landmark in implant surgery.

## 1. Introduction

Dental implant therapy has become one of the most commonly performed minor surgical procedures for the rehabilitation of missing teeth. Successful implant placement requires not only careful consideration of the patient’s restorative needs but also a thorough understanding of the anatomical limitations in the target region [1]. It is essential to be aware of the location and interrelationship of neurovascular structures, canals, and sinuses in the area where the implant will be placed, especially in relation to tissues involved in oral functions, such as tongue muscles and salivary glands [2].

The posterior region of the mandible is often utilised in dental implant surgery due to its higher bone density and volume. However, this region also contains several critical anatomical structures [3]. Two major anatomical features significantly influence implant planning in this region: These are the mandibular canal (MC), which contains the inferior alveolar nerve, and the submandibular fossa, which contains the salivary gland and dense neurovascular structures. Injury to the inferior alveolar nerve within the MC may result in paresthesia, anaesthesia, or even permanent nerve damage, which can significantly impact the patient’s quality of life [4]. The submandibular gland is just below and slightly posterior to the inferior border of the mandible and is surrounded by several important anatomical structures, including nerves, blood vessels, and lymph nodes. These must be carefully considered during surgical interventions [5]. The identification of consistent and stable anatomical landmarks is of high clinical value in order to minimise surgical complications. The mylohyoid ridge (MR), also known as the linea mylohyoidea or linea obliqua interna, is a distinct bony prominence located on the inner surface of the mandible, serving as the attachment site for the mylohyoid muscle. It is typically found underneath the premolar and molar teeth in the posterior region of the mandible. This structure is both clinically palpable and radiologically visible, making it a valuable anatomical landmark during surgical procedures [6]. Notably, the mylohyoid ridge is part of the basal bone of the mandible; it is unaffected by alveolar bone resorption and retains its position regardless of tooth loss [7,8,9]. Therefore, the MR represents a clinically significant anatomical landmark that can aid in identifying both bone density and safe zones for surgical intervention in the posterior mandibular region. Evaluating anatomical MR morphology using CBCT imaging helps clinicians to assess the difficulty level of implant placement more accurately. This, in turn, allows for better prediction of the potential need for bone augmentation procedures before or during implant placement [10].

The MR is also located at the upper border of the fossa, where the submandibular gland is located. The deepest point on the lingual surface of the submandibular fossa is called the lingual concavity (LC), which is an important anatomical point in terms of the risk of perforation during implant surgery. The most commonly reported complications of implant surgery in this area are lingual cortical perforation and inferior alveolar nerve injury [11,12]. Understanding the relationship between the mylohyoid ridge (MR) and lingual concavity (LC) helps avoid lingual bone perforation during implant placement.

In conclusion, although the MR has been acknowledged in the literature as a radiographically stable anatomical landmark, detailed morphometric analysis of its spatial relationship with the LC and the MC remains limited. A better understanding of these relationships is essential to reduce the risk of complications such as lingual cortical plate perforation and inferior alveolar nerve injury during implant placement. Therefore, we hypothesise that the positional relationships among the MR, LC and MC demonstrate consistent morphometric patterns across individuals, thus serving as reliable landmarks for implant surgery planning. The aim of this study was to determine the position of the MR and LC within the mandible and to evaluate their relationship with the MC and the submandibular fossa, in order to provide anatomical guidance for surgical procedures in this region.

## 2. Materials and Methods

This study was approved by the Non-Interventional Clinical Research Ethics Committee of Istanbul Medipol University (Decision No: 10840098-772.02-5438, dated 1 September 2023). In this retrospective study, 200 CBCT scans, comprising 98 left and 102 right hemimandibular sides, were evaluated. The dataset comprised 92 male patients and 108 female patients, aged between 27–68 years and 33–74 years, respectively. The scans were obtained from the archives of a private dental clinic. They were originally acquired for various diagnostic purposes, including endodontic treatment planning and implant surgery evaluation. For the purposes of this study, only patients who were scheduled for implant treatment were considered for inclusion.

Although many patients had partial edentulism in the posterior mandible or other regions, only those with intact mandibular first and second molars were included to ensure standardization and reproducibility. Since the MR is a continuous bony structure rather than a single point, the region between the first and second molars was chosen as a consistent and clinically relevant reference for morphometric measurements.

Other inclusion criteria for CBCT images were:

- No history of jaw fractures;
- No previous bone grafting;
- Absence of pathological lesions in the region of interest;
- Clear visibility and no motion artifacts on the CBCT images.

**Figure 1:**
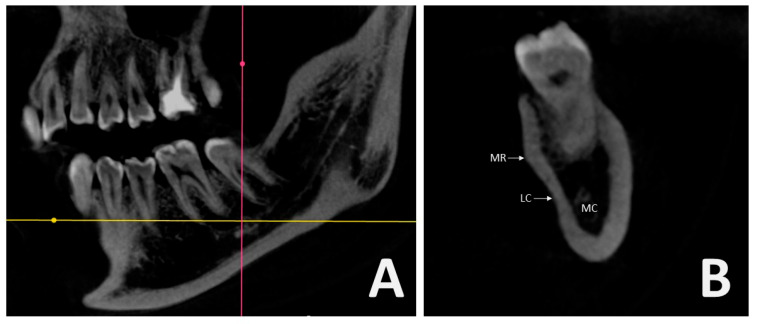
Sagittal and cross-sectional hemimandible on CBCT: (**A**) Point between the second molar and first molar tooth root in the sagittal image; (**B**) Cross-sectional images showing mylohyoid ridge (MR), lingual concavity (LC), mandibular canal (MC).

All scans were obtained using a single CBCT unit (Orthopantomograph OP300 Model, Instrumentarium Dental, Tuusula, Finland) with operating parameters of 5.0 mA and 90 kV, providing an 8 × 15 cm field of view and a voxel size of 0.25 mm. Digital Imaging and Communications in Medicine (DICOM) files were extracted from the CBCT datasets and analyzed using RadiAnt DICOM Viewer 2020.2 (64-bit trial version, Medixant, Poznan, Poland) software. Anatomical structures including the MR, LC, and MC were identified and assessed in the region between the roots of the first and second mandibular molars (Figure 1A,B).

**Figure 2:**
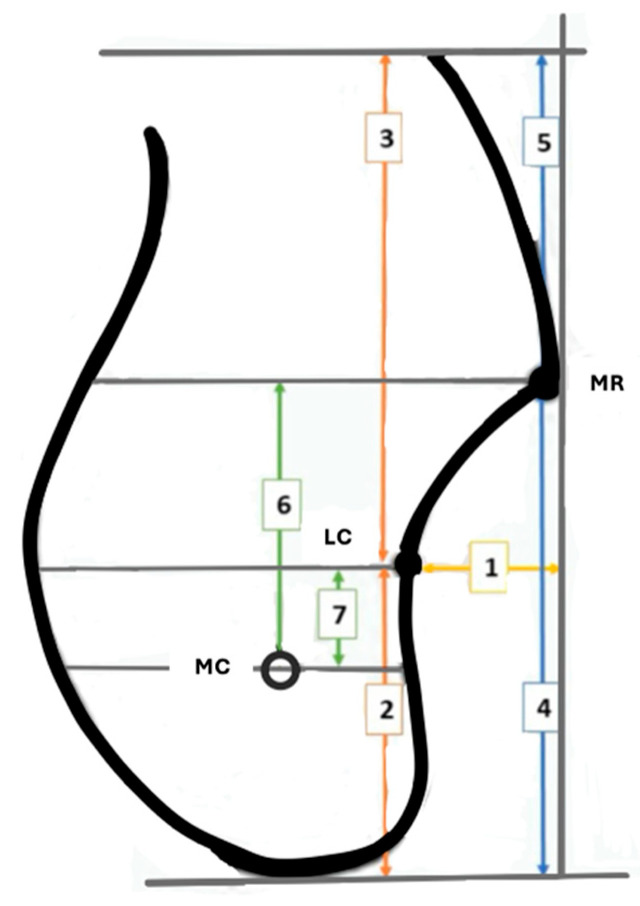
Diagrammatic representation of reference points: (1) LC horizontal depth; (2) LC height; (3) LC depth; (4) MR height; (5) MR depth; (6) MR-MC; (7) LC-MC. Mylohyoid ridge (MR), lingual concavity (LC), mandibular canal (MC).

The following parameters of the posterior mandibular region were evaluated using the measurement tool in the RadiAnt radiological examination program on cross-sectional images (Figure 2):

LC horizontal depth (submandibular fossa depth): The horizontal distance between a line descending vertically from the MR and the LC was measured.

LC height: The vertical distance between the LC and the lower edge of the mandible was measured.

LC depth: The vertical distance between the LC and the upper edge of the mandible was measured.

MR height: The vertical distance between the MR and the lower edge of the mandible was measured.

MR depth: The vertical distance between the MR and the upper edge of the mandible was measured.

MR-MC distance: A parallel line was drawn through the midpoint of the MC. Another line was drawn parallel to the first line from the MR. Then the vertical distance between the two lines was measured.

LC-MC distance: A parallel line was drawn through the midpoint of the MC. Another parallel line was drawn from LC to the first line, and the vertical distance between the two lines was measured.

Statistical analyses were conducted using SPSS software (version 26.0, IBM Corp., Armonk, NY, USA). The normality of data distribution was assessed both visually (via histograms and probability plots) and analytically using the Kolmogorov–Smirnov test. Descriptive statistics were presented as mean ± standard deviation, minimum, and maximum values for continuous variables with normal distribution. For ordinal and nominal variables, frequency and percentage (%) values were reported. Measurements were compared between groups based on sex and side (right versus left) using a Student’s *t*-test for normally distributed variables and a Mann–Whitney U test for variables that did not show normal distribution. The Pearson correlation coefficient was used to evaluate relationships between continuous variables. Correlation strength was classified as follows: Strong: r > 0.750, moderate: r = 0.500–0.750, weak: r < 0.500.

## 3. Results

Measurements were performed on CBCT images of 200 hemimandibular sides, comprising 98 left (49%) and 102 right (51%). The study group consisted of 92 male (46%) and 108 female (54%) individuals.

Mean values (in mm), standard deviations, and minimum and maximum values for each of the anatomical parameters measured in all cases (*n* = 200) are shown in Table 1.

**Figure 3:**
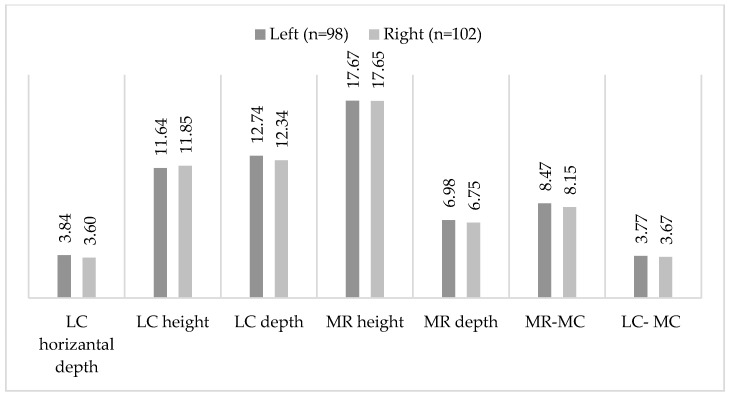
Diagram of mean measured variables on the left and right sides.

Table 2 and Figure 3 show the measurement values (in mm) for the left and right sides and a comparison between the two sides. All parameters showed no significant difference between the sides (*p* > 0.05).

**Figure 4:**
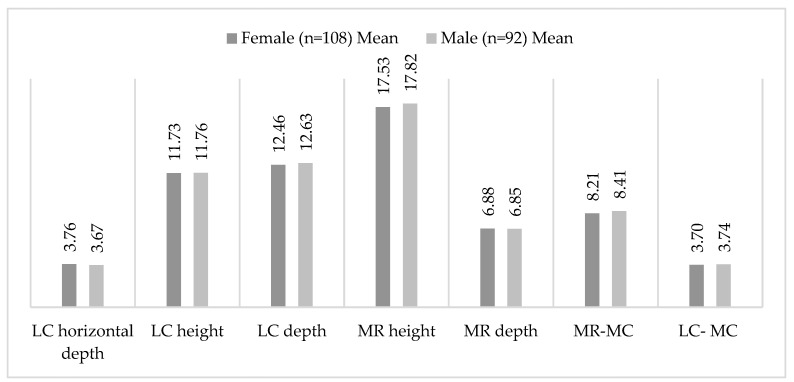
Diagram of mean measured variables in males and females.

Table 3 and Figure 4 show the measurement values (in millimetres) for the genders. No statistically significant differences in the measured parameters were observed based on sex (*p* > 0.05).

MR-MC and LC-MC distance were positively correlated in all cases (right: r = 0.200 *p* = 0.044, left: r = 0.317 *p* = 0.001, female: r = 0.289 *p* = 0.002, male: r = 0.232 *p* = 0.026). MR-MC distance and LC height were positively correlated in all cases (right: r = 0.270 *p* = 0.006, left: r = 0.308 *p* = 0.002, female: r = 0.309 *p* = 0.001, male: r = 0.257 *p* = 0.014). In addition, MR-MC distance and MR height were positively correlated in all cases (right: r = 0.259 *p* = 0.009, left: r = 0.368 *p* = 0.000, female: r = 0.361 *p* = 0.000, male: r= 0.260 *p* = 0.012).

MR depth and LC height were positively correlated in all cases (right: r = 0.376 *p* = 0.000, left: r = 0.390 *p* = 0.000, female: r = 0.356 *p* = 0.000, male: r = 0.405 *p* = 0.000). MR depth and LC depth were also positively correlated in all cases (right: r = 0.809 *p* = 0.000, left: r = 0.803 *p* = 0.000, female: r = 0.807 *p* = 0.000, male: r = 0.807 *p* = 0.000).

MR height and LC height were positively correlated in all cases (right: r = 0.699 *p* = 0.000, left: r = 0.763 *p* = 0.000, female: r = 0.735 *p* = 0.000, male: r = 0.544 *p* = 0.000). MR height and LC depth also showed a positive correlation in all cases (right: r = 0.464 *p* = 0.000, left: r = 0, 582 *p* = 0.000, female: r = 0.504 *p* = 0.000, male: r = 0.544 *p* = 000).

## 4. Discussion

Although the posterior mandibular region is frequently utilised in implant surgery, the presence of critical anatomical structures increases the risk of surgical complications. Special attention must be given to the inferior alveolar nerve and the submandibular gland along with its associated neurovascular structures [6]. The inferior alveolar nerve may be injured in approximately 0.5% to 8% of surgical interventions performed in this region [11]. Damage to the inferior alveolar nerve or the mandibular canal can lead to paresthesia or numbness of the jaw and corner of the mouth [13]. Additionally, since the neurovascular bundle within the mandibular canal also supplies the teeth, sudden loss of vitality in an entire quadrant may occur [14].

Another potential complication in this region is the perforation of the submandibular fossa. In addition to the presence of the submandibular gland in this region, the submental and sublingual arteries may run close to the lingual surface [14,15].

MR extends in a posterosuperior direction along the inner aspect of the mandible. It is located at the superior border of the submandibular fossa and lies close to the mandibular canal, Tözüm et al. (2022) reported a significant correlation between MR morphology and the complexity of implant planning, particularly in the region of the first and second molars, emphasizing the need for increased clinical caution in this area [10]. The deepest point of the submandibular fossa, situated vertically below the MR, is referred to as the LC. The presence of a pronounced lingual concavity is considered a potential risk factor for cortical plate perforation during surgical procedures [16].

Nickenig et al. (2015) reported that the presence of a LC is frequently observed in the posterior mandibular region—56% at the first molar and 90% at the second molar level—and that its presence is associated with a more inferiorly positioned inferior alveolar nerve [17]. Similarly, Bayrak et al. (2017) noted that the deepest point of the LC is located at the level of the second molar [18]. In contrast, Watanabe et al. (2010) observed LC visibility in 39% of cases at the first molar and 36% at the second molar level [19]. In the present study, we selected the region between the roots of the second (M2) and first (M1) molars—where both the MR and LC are prominently visible—as the standardized measurement point to provide a single consistent reference value. Notably, a lingual concavity was observed in all cases.

In our study, the LC height was 11.74 ± 2.01 mm with no significant differences between mandibular sides or between sexes. Similarly, the vertical depth of the LC was measured 12.54 ± 3.03 mm), again with no statistically significant difference by side or sex.

Chan et al. [16] reported the LC height at the first molar level as 14.9 ± 2.5 mm and the depth as 11.7 ± 2.9 mm. As the height of the mandible typically decreases posteriorly, it is expected that our values are lower than those reported by Chan et al. [16]. 

Furthermore, unlike our study, Chan et al. [16] used the cemento-enamel junction as a reference point rather than the superior border of the mandible, which may have contributed to the differences in measurements. Moderal et al. reported the distance from the cement-enamel junction to the LC at the first molar level as 20.3 ± 3.5 mm at the first molar level [6].

To our knowledge, no previous study has evaluated the distance of the LC from both the superior and inferior borders of the mandible. In our study, a significant correlation was found between the vertical location of the LC (height and depth) and that of the MR (height and depth). In other words, a higher position of the MR on the mandible was associated with a correspondingly higher position of the LC.

In our study, the mean horizontal depth of the LC was measured as 3.72 ± 0.90 mm This value did not differ significantly between sides of the mandible or between sexes. The submandibular fossa, located inferior to the MR on the lingual surface of the mandible, corresponds to the horizontal depth of the LC. Perforation of the lingual cortical plate in this area during surgical procedures may lead to significant hemorrhage and subsequent hematoma formation, which can result in life-threatening upper airway obstruction [20,21]. Furthermore, the dimensions of the submandibular gland, which is located in this fossa, are closely related to those of the submandibular fossa. In this context, Görgün and Çağlayan (2023) reported that greater submandibular fossa depth is associated with increased mediolateral dimensions of the submandibular gland [22]. Parnia et al. (2010) classified mandibular morphology into three types based on the horizontal depth of the LC [14]. According to this classification, the risk of lingual plate perforation increases in Type II morphology (depth > 2 mm) and is even higher in Type III morphology (depth > 3 mm). Our findings, therefore, highlight the importance of evaluating submandibular fossa depth to avoid surgical complications during implant placement in the posterior mandible.

Güngör et al. evaluated the depth of the submandibular fossa in the axial plane and reported a mean value of 4.467 ± 0.801 mm [22]. Parnia et al. [14] reported the mean depth of the submandibular fossa as 2.6 ± 0.85 mm based on cross-sectional images, while Bayrak et al. [18] reported a mean depth of 2.85 ± 0.8 mm and Sumer et al. [23] reported 1.99 ± 0.94 mm. The deepest point of the fossa, according to Bayrak et al., was found to be at the level of the second molar tooth, and this depth remained constant regardless of age [14,18,23]. This finding by Bayrak et al. [18] proposes that the position of the LC remains unaffected by tooth loss that occurs with age. Consequently, it may serve as a reliable anatomical reference point for evaluating residual bone volume, a critical factor in implant surgical planning. However, it is important to note that the reference points and measurement techniques used in these studies differ from those used in our study. In previous studies, the distance between the MR and a line tangent to the lowest border of the mandible was assessed, whereas in our study, the depth was measured from a line descending vertically from the MR.

Consistent with the findings of our study, Sumer et al. [23] reported submandibular fossa depths ranging from 0.7 to 4.9 mm, while Nickenig et al. [17] observed a wider range of 0.7 to 10.1 mm, with a mean depth of 3.7 mm. These measurements were taken from a vertical line descending from the MR [17,23]. However, the anatomical regions examined in these studies differ from ours. Specifically, Sumer et al. conducted their measurements in the LC region, whereas Nickenig et al. performed theirs in the premolar and canine regions [17,23].

Madhok et al. [6] evaluated this depth at the level of the M1 and M2 tooth roots, reporting values of 3.21 mm and 4.87 mm on the left side, and 2.99 mm and 4.28 mm on the right side, respectively. While no major differences were seen between the left and right sides, a sex-based difference was noted. Specifically, males showed higher depth values at the M2 root level on the right side [6]. In a related study, Yu et al. demonstrated that the lingual concavity tends to be approximately 2 mm deeper in the M2 region compared to the M1 region and reported possible asymmetry between the left and right sides [24]. In our study, we preferred to evaluate this distance from a single point between the roots of the M1 and M2 teeth, so that clinicians could obtain a single average value for this region.

Previous studies have suggested that the dimensions of the submandibular fossa remain stable throughout life and are not influenced by dental status, such as the presence or absence of teeth [8,9]. However, Yu et al. found that the depth of the submandibular fossa was greater in dentate individuals compared to edentulous patients [24].

Nickenig et al. reported a positive correlation between the position of the MC and the depth of the submandibular fossa [17]. However, in our study, no statistically significant relationship was observed between the position of the mandibular canal and the depth of the fossa (horizontal depth of the LC). Furthermore, no correlation was found between the horizontal depth of the LC and any of the other evaluated parameters.

In our study, the distance between the LC and the MC was measured as 3.72 ± 0.87 mm, with no statistically significant differences observed between the right and left sides of the mandible or between sexes Additionally, a significant correlation was found between the LC–MC and MR-MC distance. To the best of our knowledge, this is the first study to evaluate the LC–MC distance. We propose that the proximity of the LC to the MC may be a valuable anatomical parameter for clinicians, as it provides insight into the bone volume available, which is critical for surgical planning and implant placement.

In our study, the mean MR height was 17.66 ± 2.60 mm. This distance represents the distance from the MR to the lower edge of the mandible, and did not differ between the left and right sides of the mandible or between sexes.

The mean MR depth, defined as the distance from the MR to the superior border of the mandible, was 6.87 ± 2.38 mm. Similar to the height, MR depth did not show statistically significant differences between sides or between sexes. Pietrokovski et al. reported MR heights ranging between 20–46 mm and depths between 0–10 mm in dry edentulous mandibles [9].

In our study, the mean vertical distance between the MR and the MC was 8.30 ± 2.00 mm. This measurement did not significantly differ between sides of the mandible or between sexes. This parameter reflects the vertical position of the MC relative to the MR and may be a useful guide in estimating the location of the MC during surgical procedures. Furthermore, the sum of this measurement and the available residual alveolar bone height can provide an estimate of the total bone height available for implant placement in the posterior mandible [6]. However, it should be noted that significant anatomical differences exist in the course, length and branching pattern of the mandibular canal, which may affect the position of the inferior alveolar nerve and related blood vessels [4]. The most common mandibular canal variations are the bifid and trifid canals, with a prevalence of 18.87% and 1.3%, respectively, which may result from abnormal nerve canal fusion [25].

Madhok et al. reported this distance to be 4 mm and 5.5 mm at the M1 and M2 roots, respectively, with no significant differences between sides or sexes [6]. The discrepancy between our findings and those of Madhok et al. [6] may be attributable to methodological differences or ethnic and racial anatomical variation among the study populations.

In our study, MR-MC distance demonstrated a positive correlation with the positional parameters of both the LC and MR, including their respective heights and depths, across all subgroups. This finding is consistent with the results reported by Madhok et al. (2022) [6], who observed that as the LC descends, the MC is positioned more inferiorly relative to the MR. Supporting these findings, our results also demonstrate that as both the LC and MR shift inferiorly, the MC is correspondingly positioned at a lower level.

## 5. Conclusions

To summarize, our study found that the position of the MR and its relationship with the MC were symmetrical on both sides of the mandible, with no significant sex differences. Similarly, the position of the LC—in terms of height and depth—and its relationship with the submandibular fossa (horizontal depth) also exhibited bilateral symmetry without sex-based variation. Furthermore, a positive association was observed between the vertical positions of the MR and LC. Likewise, the MC position was found to correlate with both the MR and LC positions; specifically, when the MR and LC were located more superiorly, the MC was also positioned higher within the mandibular body. However, no significant association was found between the depth of the submandibular fossa and the positions of the MR, LC, or MC.

### Study Limitations

This study has several limitations that should be acknowledged. First, all morphometric measurements were performed by a single examiner, which may introduce observer bias despite standardized measurement protocols. Intraobserver reliability was not statistically evaluated through repeated measurements, which limits the assessment of measurement repeatability. Second, the relatively small sample size may limit the generalizability of the findings to broader populations. Future studies with larger cohorts are recommended to validate and expand upon these findings. Third, as the study included only cases with intact first and second mandibular molars to ensure measurement standardization, the findings may not fully represent anatomical variations in fully edentulous mandibles. Future research incorporating edentulous cases could provide further insight into changes associated with bone resorption and their implications for implant planning.

## Figures and Tables

**Table 1:** Means of measured values for all cases.

Total (*n* = 200)	Mean ± SD	Min–Max
**LC horizontal depth**	3.72 ± 0.90	1.87–6.33
**LC height**	11.74 ± 2.01	7.72–15.90
**LC depth**	12.54 ± 3.03	6.24–19.10
**MR height**	17.66 ± 2.60	10.80–23.10
**MR depth**	6.87 ± 2.38	2.88–12.10
**MR-MC**	8.30 ± 2.00	3.01–14.30
**LC-MC**	3.72 ± 0.87	2.03–6.56

**Table 2:** Mean values of the measured variables on the right and left sides.

	Left (*n* = 98)		Right (*n* = 102)		*p* Value
	Mean ± SD	Min–Max	Mean ± SD	Min–Max	
**LC** **horizontal depth**	3.84 ± 0.89	2.05–5.80	3.60 ± 0.90	1.87–6.33	0.050
**LC height**	11.64 ± 1.97	7.85–15.90	11.85 ± 2.06	7.72–15.80	0.486
**LC depth**	12.74 ± 3.09	7.38–19.10	12.34 ± 2.98	6.24–19.10	0.485
**MR height**	17.67 ± 2.63	10.8–23.10	17.65 ± 2.58	10.8–22.80	0.872
**MR depth**	6.98 ± 2.38	3.21–12.10	6.75 ± 2.38	2.88–12.10	0.444
**MR-MC**	8.47 ± 2.06	3.01–14.30	8.15 ± 1.93	3.01–14.20	0.342
**LC-MC**	3.77 ± 0.87	2.03–6.02	3.67 ± 0.87	2.11–6.56	0.463

**Table 3:** Mean values of the measured variables for males and females.

	Female (*n* = 108)		Male (*n* = 92)		*p* Value
	Mean ± SD	Min–Max	Mean ± SD	Min–Max	
**LC horizontal depth**	3.76 ± 0.93	1.88–6.33	3.67 ± 0.87	1.87–5.80	0.510
**LC height**	11.73 ± 1.98	7.72–15.90	11.76 ± 2.06	7.96–15.80	0.964
**LC depth**	12.46 ± 2.97	7.49–19.10	12.63 ± 3.11	6.24–19.10	0.625
**MR height**	17.53 ± 2.65	10.8–22.80	17.82 ± 2.55	10.8–23.10	0.516
**MR depth**	6.88 ± 2.33	3.21–12.10	6.85 ± 2.46	2.88–12.10	0.841
**MR-MC**	8.21 ± 1.90	3.01–14.30	8.41 ± 2.11	3.01–14.30	0.799
**LC-MC**	3.70 ± 0.90	2.03–6.56	3.74 ± 0.83	2.25–6.56	0.72

## Data Availability

The data that support the findings of this study are not publicly available due to clinical ethics restrictions and patient confidentiality. However, access to de-identified data may be considered by the corresponding author upon reasonable request and with appropriate institutional approvals.

## Abbreviations

The following abbreviations are used in this manuscript:

MR	Mylohyoid Ridge
LC	Lingual Concavity
MC	Mandibular Canal
CBCT	Cone Beam Computed Tomography
M1	Roots of the first molars
M2	Roots of the second molars
